# Histone Demethylase JMJD3 Mediated Doxorubicin-Induced Cardiomyopathy by Suppressing SESN2 Expression

**DOI:** 10.3389/fcell.2020.548605

**Published:** 2020-09-29

**Authors:** Panxia Wang, Rui Lan, Zhen Guo, Sidong Cai, Junjian Wang, Quan Wang, Zeyu Li, Zhenzhen Li, Qianqian Wang, Jingyan Li, Zhongkai Wu, Jing Lu, Peiqing Liu

**Affiliations:** ^1^School of Pharmaceutical Sciences, Sun Yat-sen University, Guangzhou, China; ^2^School of Pharmaceutical Sciences, International Institute for Translational Chinese Medicine, Guangzhou University of Chinese Medicine, Guangzhou, China; ^3^Department of Cardiac Surgery, First Affiliated Hospital, Sun Yat-sen University, Guangzhou, China; ^4^National and Local United Engineering Lab of Druggability and New Drugs Evaluation, Sun Yat-sen University, Guangzhou, China; ^5^Guangdong Provincial Engineering Laboratory of Druggability and New Drugs Evaluation, Guangzhou, China

**Keywords:** JMJD3, histone modification, SESN2, cardiotoxicity, H3K27me3

## Abstract

Jumonji domain-containing 3 (JMJD3) protein, a histone demethylase protein, specifically catalyzes the demethylation of H3K27 (H3K27me3) and regulates gene expression. Sestrin2 (SESN2), a stress-inducible protein, protected against doxorubicin (DOX)-induced cardiomyopathy by regulating mitophagy and mitochondrial function. Here, the expression of JMJD3 was increased and that of SESN2 was decreased in both the heart samples from patients with dilated cardiomyopathy and chronic DOX-stimulation induced cardiomyopathy. Inhibition or knockdown of JMJD3 attenuated DOX-induced cardiomyocytes apoptosis, mitochondrial injury and cardiac dysfunction. However, JMJD3 overexpression aggravated DOX-induced cardiomyopathy, which were relieved by SESN2 overexpression. JMJD3 inhibited the transcription of SESN2 by reducing tri-methylation of H3K27 in the promoter region of SESN2. In conclusion, JMJD3 negatively regulated SESN2 via decreasing H3K27me3 enrichment in the promoter region of SESN2, subsequently inducing mitochondrial dysfunction and cardiomyocytes apoptosis. Targeting the JMJD3-SESN2 signaling axis may be a potential therapeutic strategy to protect against DOX-mediated cardiomyopathy.

## Introduction

Doxorubicin (DOX) is an effective broad-spectrum anthracycline antibiotic, which was commonly used in solid tumors and cancers (such as lung, breast, and severe leukemia) since the late 1960 (Pacher et al., 2002; Li et al., 2006). However, the lethal cardiotoxicity side effects of DOX limit its clinical application (Olson and Mushlin, 1990; Gao et al., 2014; Hosseini et al., 2017). By inducing mitochondrial dysfunction and increasing ROS accumulation, DOX could induce numerous cytopathological responses and even cardiomyocytes apoptosis (Wallace, 2007). DOX-induced cardiotoxicity is characterized by cardiac atrophy, cellular apoptosis, destruction of mitochondrial structure as well as depolarization of mitochondrial membrane (Hu et al., 2018). Ultimately, DOX-induced decrease of cardiac function and even cardiomyopathy (Vandenwijngaert et al., 2017). Mechanically, DOX treatment obviously activated apoptosis pathway of cardiomyocytes and induced ROS accumulation and cytochrome C release (Davies and Doroshow, 1986). Despite previous investigations (Gianni et al., 2008), the pathogenic mechanisms responsible for DOX-induced cardiomyopathy were not clear and need further exploration and the effects of drugs used to ameliorate cardiomyopathy were limited and unsatisfactory.

Histone demethylase Jumonji D3 (JMJD3) protein was also called lysine-specific demethylase 6b (KDM6b) (Salminen et al., 2014; Wang P. et al., 2018; Sui et al., 2019). As histone demethylase, JMJD3 contains Jumonji catalytic domain and C-terminal segment, which is able to catalyze the H3K27me3 to H3K27me2/1 and specifically regulate gene expression (Salminen et al., 2014). According to previous studies, JMJD3 was widely reported to be related to many diseases including cancer, inflammation, neurodegenerative diseases and cardiac hypertrophy (Burchfield et al., 2015; Zhang et al., 2019). The target genes of JMJD3 were also obviously induced by lipopolysaccharide (LPS, approximately 70%) stimulation (De Santa et al., 2009; Przanowski et al., 2014). Knockout of JMJD3 inhibited apoptotic signaling pathway (including Caspase-3) to protect against neuronal apoptosis and stroke (Zhang et al., 2018). JMJD3 overexpression activated the early disease-relevant inflammation via demethylation of H3K27me3 (Wang P. et al., 2018). Our previous study has revealed that JMJD3 played a role in pathological cardiac hypertrophy via regulating H3K27me3 level in the promoter region of β-myosin heavy chain (β-MHC) (Guo et al., 2018). In this study, we further explored the effects of JMJD3 on DOX-induced cardiomyopathy and the potential mechanisms.

Sestrins (SESNs) protein family are a group of stress-inducible metabolic proteins that highly evolutionarily conserved, and have been suggested to reduce ROS generation (Liang et al., 2016). Sestrin2 (SESN2) is a member of SESNs and plays a pivotal role in physiological functions including diverse age-associated metabolic pathologies and obesity-induced pathologies (Kim et al., 2015). It was reported that SESN2 activated AMP-activated protein kinase (AMPK) and inhibited mechanistic target of rapamycin (mTOR) pathway (Kim et al., 2015). Our previous study found that SESN2 protected against DOX-induced acute cardiotoxicity via rescuing mitophagy as well as improving mitochondrial function (Wang et al., 2019). However, inconsistent with results in DOX-induced acute cardiotoxicity, we found that the transcriptional of SESN2 was depressed in dilated cardiomyopathy from human samples or following chronic Dox stimulation. In this report we detailly investigated the mechanisms on the transcriptional regulation of SESN2.

In this study, we demonstrated that the expression of histone demethylase JMJD3 were increased, while that of SESN2 were reduced in both human hearts with dilated cardiomyopathy and DOX-induced chronic cardiomyopathy model. Knockdown the expression of JMJD3 or using GSK-J4 to inhibit the function of JMJD3 effectively alleviated DOX-induced chronic cardiomyopathy and mitochondrial dysfunction. However, JMJD3 overexpression aggravated DOX-induced cardiomyopathy, which were alleviated by SESN2 overexpression. Mechanistically, JMJD3 suppressed the mRNA and protein expression of SESN2 via decreasing H3K27me3 enrichment in the promoter region of SESN2 and then induced cardiomyocytes apoptosis and mitochondrial injury. These results suggested that JMJD3 played a critical role in DOX-induced chronic cardiomyopathy by suppressing the transcription of SESN2.

## Materials and Methods

### Reagents and Antibodies

Doxorubicin (Adriamycin) hydrochloride (DOX, purity > 99%, T1020) was purchase from Target Molecule (Boston, MA, United States). GSK-J4 Hcl (S7070), a specific inhibitor of JMJD3/UTX, was purchased from Selleck Chemicals (Houston, TX, United States). Dulbecco’s Modified Eagle Medium (DMEM) and Fetal Bovine Serum (FBS) were obtained from Life Technologies (Grand Island, NY, United States). Anti-Bax (ab32503, rabbit, diluted: 1:1000), Anti-JMJD3 (ab169197, rabbit, diluted 1:1000) were from Abcam (Cambridge, MA, United States). Anti-SESN2 antibody (66297-1-Ig, Boston, MA, United States) was from Cell Signaling Technology. Anti-Caspase-3 antibody (19677-1-AP, rabbit, diluted at 1:1000) was from Proteintech group (Rosemont, IL, United States). Polyclonal Anti-Bcl-2 antibody (A0040-1, rabbit, diluted 1:1000) was purchased from Boster Biological Technology (Hubei, China). Cleaved Caspase-3 antibody (Asp75, rabbit, diluted 1:1000) was from Cell Signaling Technology (Beverly, MA, United States). Anti-α-Tubulin (T6199, mouse, diluted 1:5000) was from Sigma-Aldrich (St. Louis, MO, United States). Recombinant adenovirus vectors encoding JMJD3 (Ad-JMJD3, 10^10^ particles) and green fluorescent protein (Ad-GFP, 10^10^ particles) were obtained from Genechem (Shanghai, China). Recombinant adenovirus vectors encoding SESN2 (Ad-SESN2, 10^10^ particles) were obtained from Vigene Biosciences (Shandong, China).

### Human Heart Samples With Dilated Cardiomyopathy

All patients or the family of prospective heart donors have written informed consent prior to participation. Approval was obtained from the human ethics committee of First Affiliated Hospital of Sun Yat-sen University and the approval number was No. [2017]157. The human heart samples with dilated cardiomyopathy were collected from 7 patients undergoing heart transplantation. Three healthy heart samples were obtained from prospective multi-organ donors, which did not exhibit cardiovascular pathology but were unable to be transplanted due to technical reasons.

### Animal Experiment

All of our animal experimental procedures were strictly conformed to the Guide for the Care and Use of Laboratory Animals (NIH Publication No. 85-23, revised 1996) and approved by the Experimental Animal Center of Sun Yat-sen University (Guangzhou, China). The approval number was SYSU-IACUC-2019-000310. C57BL/6J mice (male, weighing 15–20 g, SPF grade, Certification No. 44008500018759) and Sprague-Dawley rats (SD, male, weighing 200–230 g, SPF grade, Certification No. 44008500016538) were supplied by the Experimental Animal Center of Sun Yat-sen University (Guangzhou, China). Dox was treat to rats or mice for 3 weeks. DOX was administrated to rat at a cumulative dose 20 mg/kg or to C57BL/6J mice at a cumulative dose 28 mg/kg for four independent equal dosage (at 1st, 5th, 9th, and 13th day). C57BL/6J mice were pretreated with GSK-J4 (10 mg/kg/day, *i.p.*) for 1 week and then co-treated with DOX. Animals in the control group received equal volume of normal saline (NS).

### Intramyocardial Injection of Recombinant Adenovirus of JMJD3

Sprague-Dawley rats (male, weighing 200–230 g) were randomly divided into Ad-JMJD3 group and Ad-GFP group. Sodium pentobarbital (45 mg/kg, Merck, *i.p.*) was used for anesthesia. Subsequently, rats were endotracheally intubated non-invasively connecting to respiratory machine and the heart was exposed at the left third to fourth intercostal space. Ad-GFP (10^10^ particles) or Ad-JMJD3 (10^10^ particles) with the volume of 200 μL were injected into 6 sites around the heart of left ventricular walls (each site at the depth of 1–2 mm) by using sterile disposable insulin syringe (25-gauge, Bayon, Germany). After the operation, thoraces and incision were closed. Gentamicin was given to prevent infection and meloxicam (2 mg/kg, s.q) was administrated to rat for 1 week to relieve pain.

### Echocardiographic and Morphometric Analysis

Two-dimensionally guided M-mode echocardiography was detected by Technos MPX ultrasound system (ESAOTE, SpAESAOTE SpA, Italy). The method was reported in our previous study (Zhou et al., 2006). Rats or mice were lied on a heating pad to maintain the body temperature at 37°C after anesthetized with 4% (v/v) isoflurane. After M-mode recording, basic cardiac function parameters were analyzed as followed: Cardiac Output (CO, mL/min), Ejection Fraction (EF,%), Fractional Shortening (FS,%), Stroke Volume (SV, μL), Heart Rate (HR, BPM), Left Ventricular end-diastolic Anterior Wall thickness (LVAW-d, mm), Left Ventricular end-systolic Anterior Wall thickness (LVAW-s, mm), Left Ventricular end-diastolic Posterior Wall thickness (LVPW-d, mm), Left Ventricular end-systolic Posterior Wall thickness (LVPW-s, mm), Left Ventricular end-diastolic Internal Dimension (LVID-d, mm), and Left Ventricular end-systolic Internal Dimension (LVID-s, mm).

At the end of all experiments, animals were anesthetized by sodium pentobarbital (45 mg/kg, Merck, *i.p.*) and hearts were removed with 10% potassium chloride solution injection to arrest the heart. The heart was washed in ice-cold PBS. Histological cross (5-μm-trick) of the heart tissues were embedded in paraffin or stained with Picrosirius red (PSR), Masson, Hematoxylin-eosin (HE), Immunohistochemistry (IHC) for morphometric measurement.

### Primary Culture of Neonatal Rat Cardiomyocytes (NRCMs)

Previous studies in our laboratory have described the method for primary culture of NRCMs (Cai et al., 2012; Yu et al., 2013; Lu et al., 2016; Li et al., 2018). SD rat (1 to 3- day-old) were anesthetized. The hearts were rapidly removed, washed in ice-cold phosphate buffered saline (PBS) buffer and then chopped into 4–6 pieces. Subsequently, heart tissues were digested with 0.08% trypsin solution at 37°C for 10-14 times (4 min 30 s each time) until all tissues were disappeared. By centrifugating at 1500 g for 6 min, all cells were collected. For the differential attachment between cardiomyocytes and fibrosis, suspension with cardiomyocytes were collected following cultured for 1 h at 37°C with 5% CO_2_ atmosphere. Finally, the cells were plated at a density of 1 × 10^6^ per well onto culture dishes (35 mm) in DMEM with 10% FBS and 5-bromodeoxyuridine (BrdU, 0.1 mM). Cell culture media was refreshed 24 h later. DOX at different concentrations (0.1, 0.5, and 1 μM) was treated to NRCMs at the indicated time points. Accordingly, equal volume of DMEM (without DOX) was administrated to control group.

### Quantitative Real-Time Polymerase Chain Reaction (qRT-PCR)

Total RNA was extracted from NRCMs or heart tissues by Trizol reagent (Invitrogen, Carlsbad, CA, United States). The two-step RT Kit obtained from Thermo Fisher Scientific (Rockford, IL, United States) was used to reversely transcribe the RNA into first cDNA. As previously described (Wang L. et al., 2018), the mRNA levels of target genes were measured by PCR analyses with SYBR Green Quantitative PCR Kit (TOYOBO, Japan). Rat-specific primers for JMJD3, SESN2 and β-actin were synthesized by Sangon Biotech Co., Ltd. (Shanghai, China). β-actin was used as house-keeping gene. The sequence of rat-specific primers was as follows: JMJD3, 5′- TCAGGAGAGGAAGGCCTCAG-3′ and 5′-AGCTGGGTATGGATGAGGGT-3′. SESN2, 5′-TACCTTA GCAGCTTCTGGCG-3′ and 5′-AGGTAAGAACACTGGT GGCG-3′, β-actin, 5′-TCGTGCGTGACATTAAAGAG-3′ and 5′-ATTGCCGATAGTGATGACCT-3′.

### Western Blot Analysis

Protein of NRCMs or frozen heart tissues (left ventricular) were collected by using RIPA lysis buffer (Beyotime, Jiangsu, China) supplemented with protease inhibitor cocktail (Sigma-Aldrich, St. Louis, MO, United States). The concentration of protein was measured by using a Bicinchoninic Acid Protein Assay Kit (Pierce, Rockford, IL, United States). Total protein samples were separated by SDS-PAGE gels and transferred onto PVDF membranes. 5% skim milk was used to block all membranes and first antibodies were incubated with membranes at 4°C overnight. Finally, the protein bands were detected by using a High-sigECL Western Blotting Substrate kit (Tanon, Science & Technology Co., Ltd., Shanghai, China). The band intensity was detected by automatic chemiluminescence image analysis system (Tanon-5200, Tanon, Science & Technology Co., Ltd., Shanghai, China) and analyzed with Image J software (BioRad, CA, United States).

### RNA Interference

Primary-cultured of neonatal rat cardiomyocytes were transfected with small interfering RNAs (siRNA, 100 pmol for 35 mm dishes) by using lipofectamine 2000 (Invitrogen, Carlsbad, CA, United States) for 72 h. The siRNA targeting to JMJD3 and negative control (NC) were purchased from Genepharma (Shanghai, China). The sequence of the JMJD3 siRNA or NC siRNA was listed as follows: si-JMJD3, 5′-GCCUUCAUGCGAGUAACAUTT-3 and 5′-AUGUUACUCGCAUGAAGGCTT-3′. NC, 5′-UUCUCCGAA CGUGUCACGUTT-3′ and 5′-ACGUGACACGUUCGGAGAA TT-3′. Western blotting was performed to determine the silencing efficiency for JMJD3.

### Chromatin Immunoprecipitation (ChIP)-qPCR Analysis

Firstly, formaldehyde was directly added to the media and swirled gently, and was incubated at room temperature for 10 min. Then, glycine was added and swirled gently to mix. Cells were washed with PBS three times and were lysated by 5 mL Farnham lysis buffer (including protease inhibitors). Cells were scraped and transferred into conical tubes, followed by centrifugation at 2,000 rpm for 5 min before the supernatant was removed. After that, the cell pellets were pre-chilled in liquid nitrogen. Secondly, frozen cell pellets were re-suspended in 5 mL Farnham lysis buffer containing protease inhibitors and were centrifuged at 2,000 rpm at 4°C for 5min. Then, supernatant was removed and 1 mL RIPA buffer (including protease inhibitors) was added into pellets before sonication. Thirdly, pellets were mixed with 25 μL magnetic beads and 1 mL PBS in a 1.5 mL microfuge tube, the tube was placed on the magnetic rack to remove supernatants, after washing the beads for three times by 1 mL PBS, the beads were incubated with 5 μg antibody and was gently rotated overnight. Then, Beads were washed three times before each sonicate sample was incubated with 50 μL coupled antibody on a rotator overnight. After that, supernatant was removed by the magnetic rack and beads were washed with LiCl wash buffer by 3 times. Finally, beads were incubated in a 65°C water bath for 1 h, the supernatant was collected after centrifugation of 15,000 rpm for 3 min, the supernatant was incubated in 65°C water bath overnight and the DNA fragments were purified using GeneJET PCR Purification kit (Thermo Scientific, K0702, United States). The purified DNA and specific primers for SESN2 promoter region were used to amplify target DNA. Primers for SESN2 are list in Supplementary Table S1.

### CRISPR/Cas9 sgRNA Design, Lentivirus Production, and Infection

sgRNA sequences targeting SESN2 were listed as follows: GCGGGTGGACAACCTGGCGG. Oligos corresponding to the sgRNAs were synthesized and cloned into lenti-CRISPR v2 vectors (Addgene, plasmid #52961). According to our previous study, lentiCRISPRv2-gRNA or lentiCRISPRv2, psPAX2 and pMD2.G were simultaneously co-transfected into HEK293T cell for 48 h to generate lentivirus (Wang et al., 2019). NRCMs were plated into 35 mm dishes and one milliliter of virus suspension was added into the culture medium. Six hours later, the culture medium was changed into fresh medium. Cultured for another 72 h and western blotting was performed to determine the targets deficient efficiency following.

### Statistical Analysis

Data were analysis with GraphPad Prism software and were presented as means ± SD. Statistical difference between two groups were evaluated with unpaired Student’s *t*-test. Difference analysis among various groups was performed by one-way ANOVA with Tukey’s *post hoc* test. In all cases, data were considered statistically significant with *P* value less than 0.05.

## Results

### The Expression of JMJD3 and SESN2 in Human Dilated Cardiomyopathy and DOX-Induced Chronic Rat Cardiotoxicity Model

**FIGURE 1:**
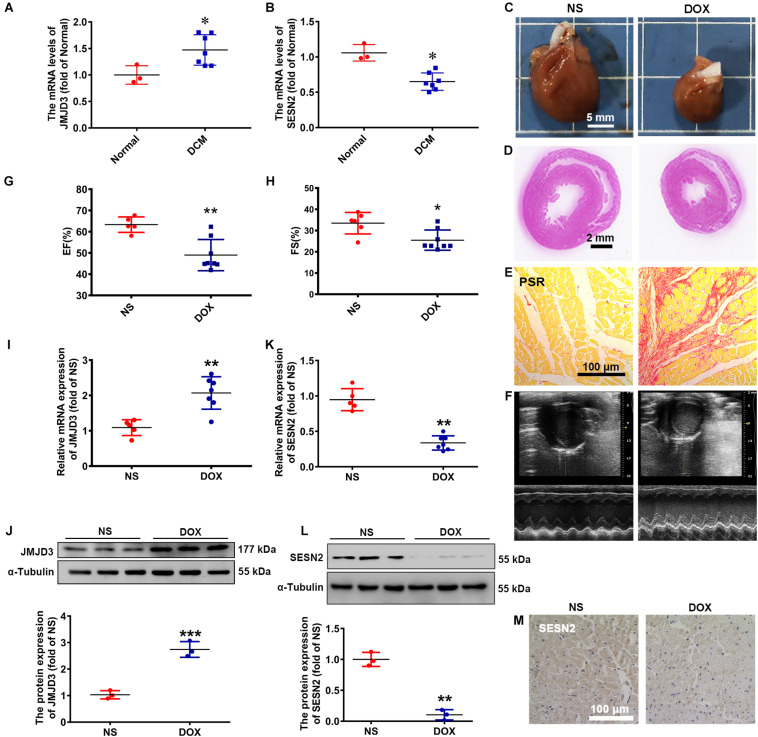
The expression of JMJD3 and SESN2 in human dilated cardiomyopathy and DOX-induced chronic rat cardiomyopathy model. **(A,B)** The mRNA levels of JMJD3 and SESN2 were measured in the human dilated cardiomyopathy, *n* = 3 in Normal group, *n* = 7 in DCM group. SD rats were treated with DOX (accumulate dose 20 mg/kg) for 3 weeks or equal volume of normal saline (NS), *n* = 6 in NS group, *n* = 9 in DOX group. **(C)** The morphologic changes of gross hearts, scale bar = 5 mm. **(D,E)** Pathological changes of hearts tissues were detected by H&E staining (scale bar = 2 mm) and PSR staining (scale bar = 100 μm). **(F)** Echocardiographic graphs. **(G,H)** EF (%) and FS (%) were analyzed. **(I,K)** The mRNA expression of JMJD3 and SESN2 were measured in the rat hearts by qRT-PCR. **(J,L)** The protein expression of JMJD3 and SESN2 were detected by Western blot. **(M)** The protein level of SESN2 was measured in by IHC, scale bar = 100 μm. Data were presented as the mean ± SD. *p < 0.05, **p < 0.01, ***p < 0.001 vs. Normal group or NS group. DCM, dilated cardiomyopathy; DOX, doxorubicin; EF, ejection fraction; FS, fractional shortening; HE, hematoxylin-eosin; i.p., intraperitoneally injection; IHC, immunohistochemistry; JMJD3, Jumonji domain-containing 3; NS, normal saline; PSR, Picro Sirius Red; SESN2, Sestrin2; SD, Sprague-Dawley.

To explore the expression changes of JMJD3 and SESN2 in dilated cardiomyopathy (DCM), we used heart samples from 7 clinic patients with cardiomyopathy and 3 healthy controls. The mRNA level of JMJD3 was increased in human heart tissues with DCM (Figure 1A), while that of SESN2 was decreased (Figure 1B).

Doxorubicin was widely used to induce dilated cardiomyopathy models *in vivo* and *in vitro* for the obvious cardiotoxicity side effects (Shi et al., 2011; Carvalho et al., 2014). In this study, DOX (20 mg/kg, *i.p.*) was used to stimulate rat for 3 weeks and induce chronic cardiomyopathy. Gross morphologic examination showed that the hearts in DOX group were significantly smaller than that of control group (Figures 1C,D). Results from hematoxylin-eosin (HE) staining, Picro Sirius Red (PSR) staining and Masson staining showed that cardiomyocytes in DOX group were apparently disorganized with increased extracellular matrix and fibrosis, and decreased myocyte diameter and ventricular wall thickness (Figures 1D,E and Supplementary Figures S1A–C). The heart weight to tibia length (HW/TL, mg/mm) was lower following DOX stimulation, although without significant difference in the ratio of heart weight/body weight (HW/BW, mg/g) (Supplementary Figures S1D,E). Echocardiographic results revealed that DOX significantly decreased ejection fraction (EF,%), fractional shortening (FS,%), cardiac output (CO, mL/min), stroke volume (SV, μL), heart rate (HR, BPM), left ventricular internal dimension (LVID, mm), left ventricular anterior wall thickness (LVAW, mm), and left ventricular posterior wall thickness (LVPW, mm) (Figures 1F–H and Supplementary Figures S1F–N). These results indicated that we successfully established DOX-induced chronic cardiomyopathy model.

Subsequently, the expression of JMJD3 and SESN2 were, respectively, measured. Consistent with results from human samples, both the mRNA and protein levels of JMJD3 were significantly elevated in DOX group (Figures 1I,J). Conversely, the expression of SESN2 was significantly inhibited in DOX-induced chronic cardiomyopathy model (Figures 1K–M and Supplementary Figure S1P). All these results indicated that the expression of JMJD3 and SESN2 were in the opposite direction during DOX chronic stimulation.

### The Expression of JMJD3 and SESN2 in DOX-Induced Chronic Cardiotoxicity

**FIGURE 2:**
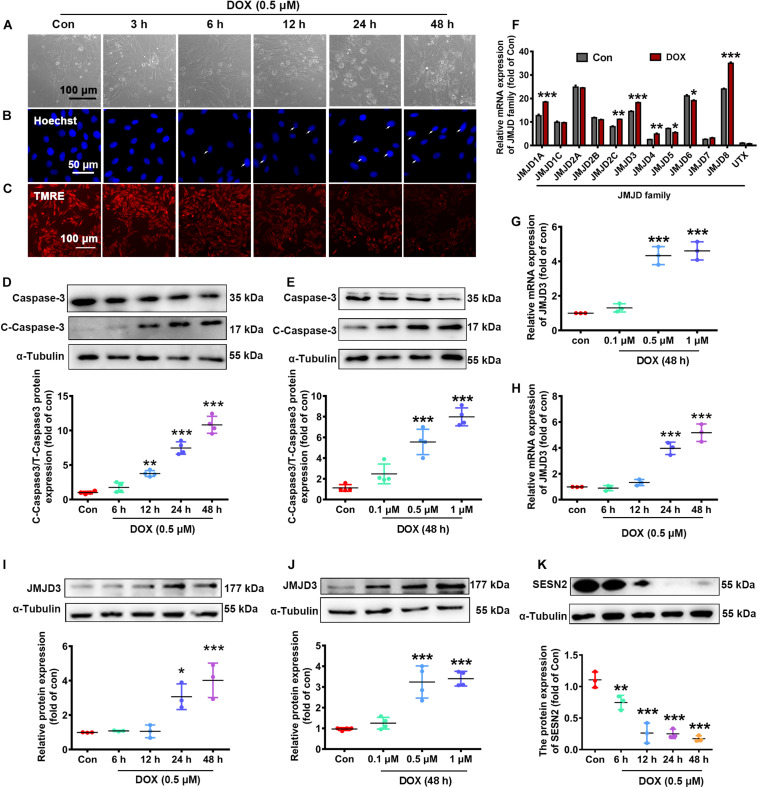
The expression changes of JMJD3 and SESN2 with chronic DOX-induced cardiotoxicity in NRCMs. NRCMs were incubated with 0.5 μM DOX for indicated time points or with different concentrations of DOX (0.1, 0.5, and 1 μM) for 48 h. **(A)** Cell morphology of cardiomyocytes was observed by using a light microscopy, scale bar = 100 μm. **(B)** Hoechst-staining was used to observe chromatin condensation (indicated by arrows), scale bar = 100 μm. **(C)** Depolarization of mitochondrial membrane potential was detected by TMRE staining, scale bar = 100 μm. **(D,E)** The protein expression of cleaved caspase3 were measured by Western blot. **(F)** The mRNA levels of JMJD family were determined by RNA-Seq analysis. **(G,H)** The mRNA level of JMJD3 was measured by qRT-PCR. **(I–K)** The protein levels of JMJD3 and SESN2 were measured by Western blot. Data were presented as the mean ± SD. *p < 0.05, **p < 0.01, ***p < 0.001 vs. Con group. *n* = 3. DOX, doxorubicin; NRCMs, primary-cultured of neonatal rat cardiomyocytes; TMRE, tetramethyl rhodamine methyl ester; JMJD, Jumonji domain-containing; JMJD3, Jumonji domain-containing 3; SESN2, Sestrin2.

We further validated the expression changes of JMJD3 and SESN2 *in vitro*. NRCMs were incubated with DOX at different doses (0.1, 0.5, and 1 μM) for different time (1, 3, 6, 12, and 24 h). As shown in Figures 2A–C and Supplementary Figures S2A,B, DOX (0.5 μM) time-dependently increased cardiomyocyte loss (Figure 2A and Supplementary Figure S2A), nuclear condensation (Figure 2B) and depolarization of mitochondrial membrane potential (Figure 2C and Supplementary Figure S2B) as indicated by light microscopy, Hoechst staining and Tetramethyl rhodamine methyl ester (TMRE) staining. Additionally, the expression of cleaved caspase3 and the ratio of BAX/Bcl-2 were time-dependently induced following DOX (0.5 μM) stimulation (Figure 2D and Supplementary Figure S2C). Furthermore, DOX stimulated for 48 h increased the expression of cleaved caspse3 and the ratio of Bax/Bcl-2 in a dose-dependent manner (Figure 2E and Supplementary Figure S2D). There results indicated that Dox (0.5 μM) stimulated for 48 h could induce chronic toxicity of cardiomyocytes and was used for the following experiment.

The transcriptional changes of JMJD protein family were determined by RNA-Sequence analyses. The results showed that the mRNA levels of several subtypes (including JMJD1A, JMJD2C, JMJD3, JMJD4, JMJD5, JMJD6, and JMJD8) were significantly changed following DOX stimulation (0.5 μM, 48 h) (Figure 2F). The mRNA level of JMJD3 was further confirmed by qPCR in DOX-treated NRCMs. Results of Figures 2G,H showed that the mRNA of JMJD3 were increased following DOX-stimulation in a time-dependent and dose-dependent manner. Consistently, the protein level of JMJD3 was significantly increased following DOX stimulation in the same manner (Figures 2I,J). On the contrary, the protein of SESN2 was significantly decreased with chronic DOX stimulation in NRCMs (Figure 2K).

The above results indicated a strong association between JMJD3, SESN2 and cardiomyopathy.

### Inhibition or Knockdown of JMJD3 Attenuated DOX-Induced Chronic Cardiotoxicity in NRCMs

**FIGURE 3:**
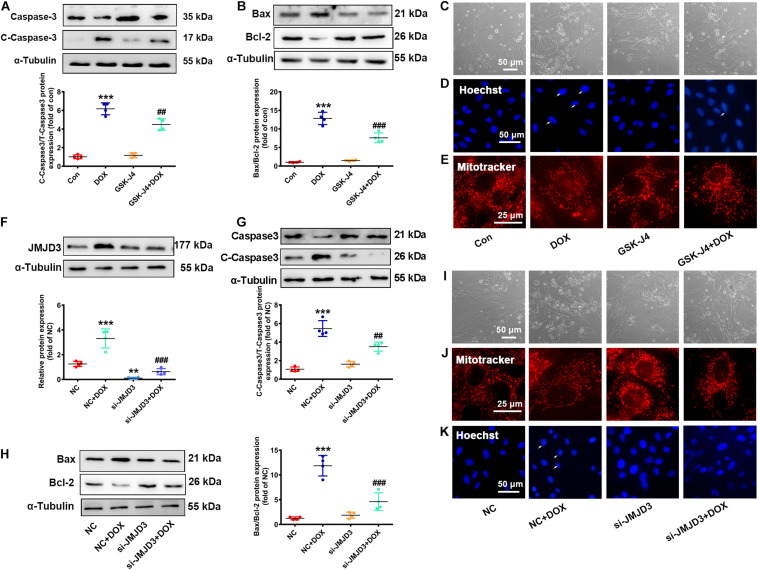
Inhibition or knockdown of JMJD3 attenuated DOX-induced cardiomyopathy. NRCMs were co-treated with GSK-J4 (5 μM, a novel and selective inhibitor of JMJD3) and DOX (0.5 μM) for 48 h. **(A,B)** Protein changes of cleaved caspase3 and BAX/Bcl-2 were measured by Western blot. **(C)** Cell morphology of cardiomyocytes was observed by a light microscopy, scale bar = 50 μm. **(D)** Chromatin condensation was indicated by Hoechst-staining (indicated by arrows), scale bar = 50 μm. **(E)** The mitochondrial structure changes of cardiomyocytes was observed by mitotracker Red staining, scale bar = 25 μm. **(F)** Endogenous JMJD3 was knocked down by using small interfering RNAs (siRNA) targeting in NRCMs. **(G,H)** Protein expression of cleaved caspase3 and BAX/Bcl-2 were analyzed by Western blot. **(I)** Cell morphology of NRCMs was observed by a light microscopy, scale bar = 50 μm. **(J)** The mitochondrial structure of cardiomyocytes was observed by mitotracker Red staining, scale bar = 25 μm. **(K)** Chromatin condensation was observed by Hoechst-staining (indicated by arrows), scale bar = 50 μm. Data were presented as the mean ± SD. **p< 0.01, ***p < 0.001 vs. Con or NC group. *n* = 3. ^##^p < 0.01, ^###^*p* < 0.001 vs. DOX group or NC + DOX group. *n* = 3. DOX, doxorubicin; NRCMs, primary-cultured of neonatal rat cardiomyocytes; JMJD3, Jumonji domain-containing 3; siRNA, small interfering RNAs.

The effects of DOX on the expression of JMJD3 encouraged us to explore whether JMJD3 was involved in DOX-induced chronic cardiotoxicity. Here, NRCMs were treated with GSK-J4 (5 μM, enzyme activity inhibitor of JMJD3) (Yan et al., 2017) with DOX (0.5 μM) co-treatment for 48 h. Western blot results showed that GSK-J4 obviously attenuated DOX-induced increase in the expression cleaved caspase3 and the ratio of BAX/Bcl-2 (Figures 3A,B). GSK-J4 also alleviated DOX-induced morphological changes of cardiomyocyte, inhibition of cell viability, increased nuclear condensation, and disorganized mitochondrial structure (Figures 3C–E and Supplementary Figure S3A). However, treatment with GSK-J4 alone did not have any effects on cardiomyocytes (Figures 3A–E).

Besides, endogenous JMJD3 was silenced by using small interfering RNAs (siRNA) in NRCMs (Figure 3F). Consistently, knockout of JMJD3 in cardiomyocyte obviously attenuated DOX-induced cardiotoxic responses as indicated by the protein changes of cleaved caspase3 and BAX/Bcl-1, morphology of cardiomyocytes, cell viability, nuclear condensation and mitochondrial structure (Figures 3G–K and Supplementary Figure S3B).

The above results indicated inhibition or knockdown of JMJD3 *in vitro* effectively alleviated DOX-induced chronic cardiotoxicity.

### GSK-J4 Protected Against DOX-Induced Chronic Cardiomyopathy in Mice

**FIGURE 4:**
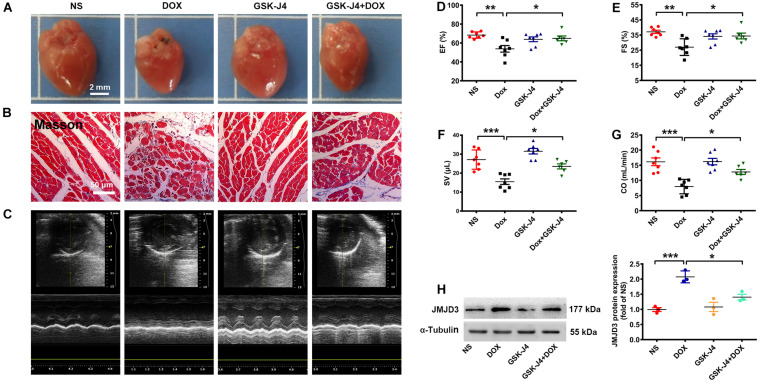
GSK-J4 improved cardiac function of DOX-induced mice cardiotoxicity model. C57BL/6J mice were treated with GSK-J4 (10 mg/kg/day, *i.p*.) or equal volume of normal saline (NS) for 7 days. After that, the mice were injected with DOX at accumulative dose 28 mg/kg or equal volume of normal saline for 3 weeks. **(A)** The morphologic changes of gross hearts, scale bar = 2 mm. **(B)** Pathological changes of hearts tissues were detected by Masson-staining, scale bar = 50 μm. **(C)** Representative echocardiographic graphs. **(D–G)** The EF (%), FS (%), SV (μL) and CO (mL/min) were measured by echocardiographic analysis, *n* = 7. **(H)** The protein changes of JMJD3 in mice hearts were measured by Western blot. Data were presented as the mean ± SD. *p < 0.05, **p < 0.01, ***p < 0.001 vs. NS group or Dox group. *n* = 7. CO, cardiac output; DOX, doxorubicin; EF, ejection fraction; FS, fractional shortening; i.p., intraperitoneally injection; JMJD3, Jumonji domain-containing 3; NS, normal saline; SV, Stroke Volume.

To further investigate the role of JMJD3 in DOX-induced cardiotoxicity *in vivo*, C57BL/6J mice were pre-treated with GSK-J4 (10 mg/kg/day, *i.p.*) for 1 week and then with DOX (accumulate dose 28 mg/kg) co-treatment for 3 weeks. GSK-J4 pre-treatment effectively alleviate DOX-induced morphologic changes and fibrosis of heart tissue (Figures 4A,B and Supplementary Figure S4C), although without significant changes in both HW/BW and HW/TL ratios (Supplementary Figures S4D,E). Echocardiographic results showed that GSK-J4 protected against DOX-induced cardiac dysfunction as indicated by increased EF, FS, SV, and CO (Figures 4C–G). Additionally, GSK-J4 treatment alone did not affect the hearts size, myocardial fibrosis and cardiac function. In line with the *in vitro* results, these results suggest that JMJD3 inhibition alleviated DOX-induced chronic cardiotoxicity *in vivo*. Besides, GSK-J4 treatment suppressed DOX-induced increase in JMJD3 protein level (Figure 4H).

### JMJD3 Overexpression Induced Cardiomyopathy *in vitro* and *in vivo*

**FIGURE 5:**
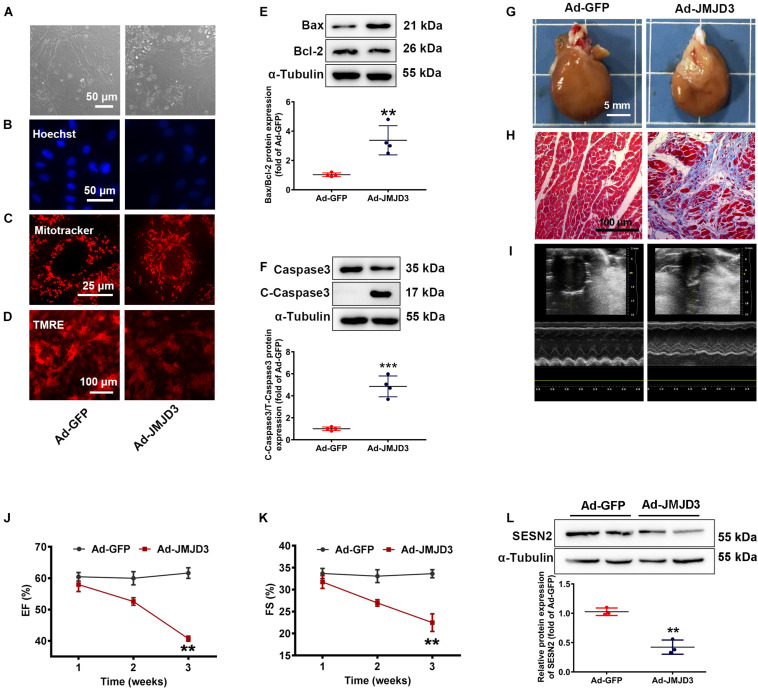
JMJD3 overexpression aggravated DOX-induced cardiomyopathy *in vitro* and *in vivo.* NRCMs were infected with Ad-JMJD3 or Ad-GFP (empty vector) for 48 h. **(A)** Morphology of cardiomyocytes was observed by a light microscopy, scale bar = 50 μm. **(B)** Chromatin condensation was shown by Hoechst staining (indicated by arrows), scale bar = 50 μm. **(C)** The mitochondrial structure of cardiomyocytes was observed by mitotracker Red staining, scale bar = 25 μm. **(D)** Depolarization of mitochondria membrane potential was detected by TMRE-staining, scale bar = 100 μm. **(E,F)** The protein changes of BAX/Bcl-2 and cleaved caspase3 were analyzed by Western blot. Moreover, Ad-JMJD3 or Ad-GFP was transduced into the rat left ventricle via intramyocardial *in situ* injection. **(G)** The morphologic changes of gross hearts, scale bar = 5 mm. **(H)** Pathological changes of hearts tissues were detected by Masson-staining, scale bar = 100 μm. **(I)** Representative echocardiographic graphs. **(J,K)** EF (%) and FS (%) were analyzed by echocardiography, *n* = 8. The cardiac function was measured in 1, 2, and 3 weeks after the transfection of Ad-JMJD3 or Ad-GFP. **(L)** The protein expression of SESN2 was measured by Western blot. Data were presented as the mean ± SD. **p < 0.01, ***p < 0.001 vs. Ad-GFP group. *n* = 3. DOX, doxorubicin; EF, ejection fraction; FS, fractional shortening; JMJD3, Jumonji domain-containing 3; SESN2, Sestrin2; NRCMs, primary-cultured of neonatal rat cardiomyocytes; TMRE, tetramethyl rhodamine methyl ester.

To further confirm the role of JMJD3 in cardiomyopathy, adenovirus vector encoding JMJD3 cDNA (Ad-JMJD3) was used to overexpress JMJD3 both *in vitro* and *in vivo*. As shown in Supplementary Figure S4A, JMJD3 was successfully overexpressed in NRCMs following Ad-JMJD3 infection for 48 h. Compared with Ad-GFP group, JMJD3 overexpression obviously induced morphologic changes of cardiomyocytes, inhibited cell viability, increased nuclear condensation, disorganized mitochondrial function as well as depolarized mitochondrial membrane potential (Figures 5A–D and Supplementary Figures S4B,C). Furthermore, overexpression of JMJD3 also increased the expression of cleaved caspase3 and the ratio of BAX/Bcl-2 (Figures 5E,F). These results indicated JMJD3 induced cardiomyocytes injury *in vitro*.

To further validate the effects of JMJD3 on cardiomyocytes, JMJD3 was overexpressed in the heart tissue via intramyocardial injection. Western blot results showed that JMJD3 was successfully overexpressed in the heart tissue (Supplementary Figure S4D). As shown in Figures 5G,H and Supplementary Figure S4E, the delivery of Ad-JMJD3 remarkably induced typical myocardial fibrosis and inflammatory infiltrates, while didn’t have much effects on heart size and weight (Supplementary Figures S4F,G). Echocardiographic analysis was performed in weeks 1, 2, and 3 after Ad-JMJD3 or Ad-GFP infection. Echocardiographic data (Figures 5I–K and Supplementary Figures S4H–K) showed that JMJD3 overexpression gradually caused a decline in cardiac function as indicated by decreased EF, FS, LVAW, and LVPW. Additionally, we further measured the changes of SESN2 protein. As shown in Figure 5L the protein level of SESN2 was significantly depressed by JMJD3 overexpression.

Both *in vitro* and *in vivo* results revealed that overexpression of JMJD3 caused cardiomyocytes apoptosis and cardiac dysfunction, which might be related to SESN2.

### JMJD3 Decreased H3K27me3 Enrichment in the Promoter Region of SESN2

**FIGURE 6:**
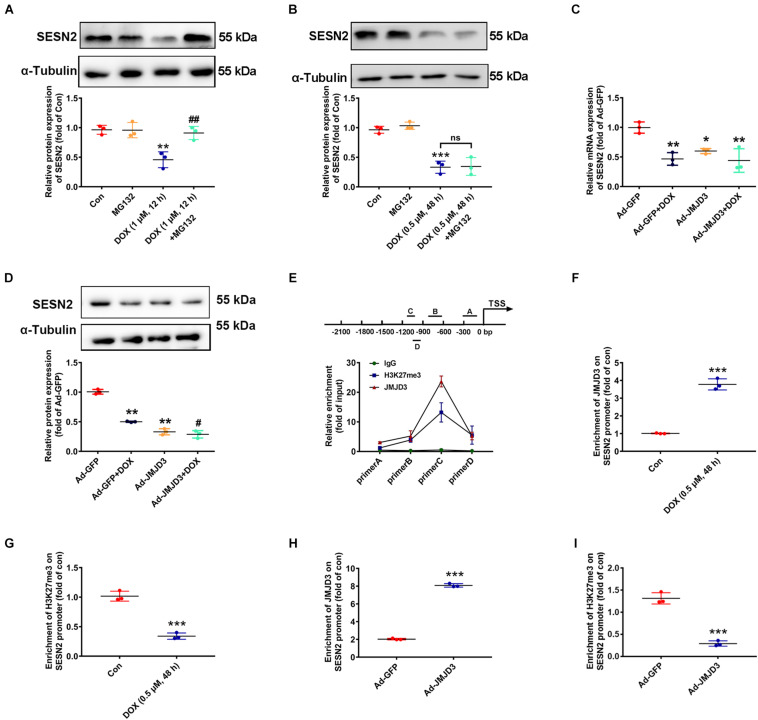
JMJD3 decreased H3K27me3 enrichment in the promoter region of SESN2. **(A)** Cardiomyocytes were co-treated with DOX (1 μM) and proteasome inhibitor MG132 (10 μM) for 12 h. **(B)** Cardiomyocytes were stimulated with DOX at relative lower concentration (0.5 μM) for longer time (48 h) and MG132 (10 μM) were co-treated. The protein expression of SESN2 were detected by western blot. **(C,D)** The mRNA and protein expression of SESN2 were measured by qRT-PCR and Western blot. Data were presented as the mean ± SD. *p < 0.05, **p < 0.01, ***p < 0.001 vs. Con or Ad-GFP group. *^#^p* < 0.05, *^##^p* < 0.01 vs. Ad-GFP + DOX group, ns means no significant differences. *n* = 3. ChIP assay was performed to detect JMJD3 and H3K27me3 enrichment on the SESN2 promoter region in NRCMs. **(E)** The immunoprecipitated protein-DNA complexes were detected with primers of SESN2. **(F,G)** The results of NRCMs treat with or without DOX (0.5 μM) for 48 h. **(H,I)** Results of cells treated with Ad-JMJD3 or Ad-GFP. Data were presented as the mean ± SD. ***p < 0.001 vs. Con group or Ad-GFP group. *n* = 3. DOX, doxorubicin; JMJD3, Jumonji domain-containing 3; SESN2, Sestrin2; ChIP, chromatin immunoprecipitation.

Subsequent experiments were performed to investigate the underlying mechanism between JMJD3 and SESN2 with chronic DOX stimulation. Inconsistent with our previous results that acute stimulation with DOX (1 μM, 12 h) had no effects on the transcription of SESN2 (Wang et al., 2019), DOX (0.5 μM, 48 h) chronically decreased the mRNA level of SESN2 (Figure 2K). Therefore, proteasome inhibitor MG132 (10 μM) was co-treated with DOX at 1 μM for 12 h or DOX at 0.5 μM for 48 h to cardiomyocytes and the protein level of SESN2 was measured. As shown Figures 6A,B, MG132 effectively reversed DOX (1 μM, 12 h)-induced decrease in the protein level of SESN2, while that was not observed in DOX (0.5 μM) stimulation for 48 h. Additionally, JMJD3 overexpression further aggravated DOX (0.5 μM, 48 h)-induced decrease in the mRNA and protein levels of SESN2 in NRCMs (Figures 6C,D).

Jumonji domain-containing 3 catalyzed demethylation H3K27me3 and regulated gene transcription (Salminen et al., 2014). Therefore, we detected the enrichment of JMJD3 and its methylation substrate H3K27me3 in the promoter region of SESN2 by using ChIP assay in NRCMs. As shown in Figure 6E, JMJD3 directly bind to the promoter region of SESN2. Furthermore, in NRCMs this binding was significantly enhanced following DOX (0.5 μM, 48 h) stimulation (Figure 6F). However, H3K27me3 enrichment in the promoter region of SESN2 was declined (Figure 6G). Moreover, similar results were also demonstrated in NRCMs with JMJD3 overexpression (Figures 6H,I). Taken together, these results partly suggest that JMJD3 suppressed SESN2 transcription in NRCMs by regulating H3K27me3 status in the promoter region of SESN2.

### Involvement of SESN2 in JMJD3-Mediated Cardiotoxicity *in vitro*

**FIGURE 7:**
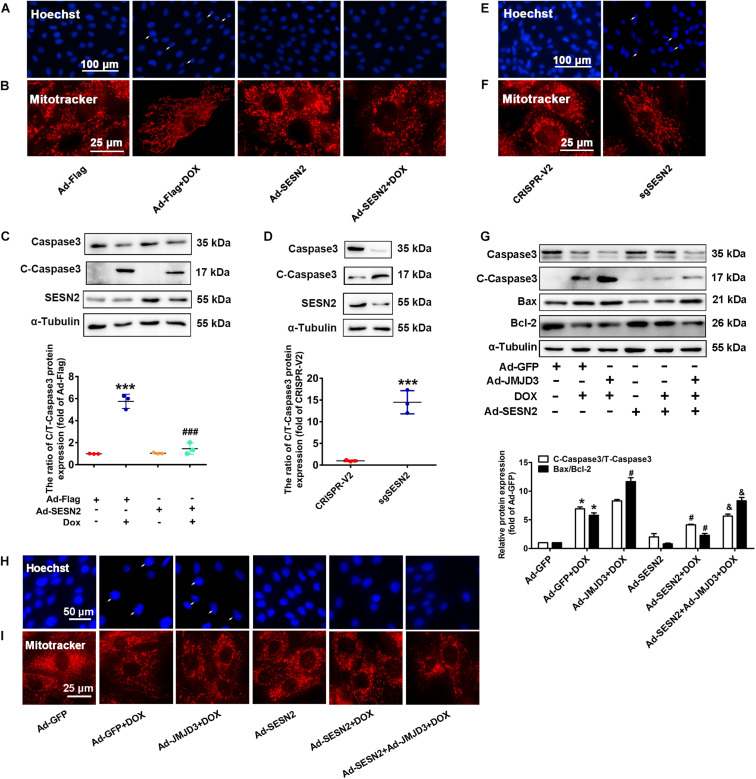
Downregulation of SESN2 contributed to cardiotoxicity induced by JMJD3 overexpression. SESN2 was overexpressed by adenovirus Ad-SESN2 or knocked out by targeting sgRNA in NRCMs with or without DOX (0.5 μM, 48 h) and JMJN3 co-treatment. **(A,E,H)** The nuclear condensation in NRCMs was observed by Hoechst staining, scale bar = 100 or 50 μm. **(B,F,I)** Mitochondrial structure changes of cardiomyocytes was indicated by Mitotracker Red staining, scale bar = 25 μm. **(C,D,G)** The protein expression of SESN2, cleaved caspase3 and BAX/Bcl2 were analyzed by Western blot. Data were presented as the mean ± SD. **(C,D)** *p < 0.05, ***p < 0.001 vs. Ad-Flag or CRISPR-V2 group. ^###^*p* < 0.001 vs. Ad-Flag + DOX group. *n* = 3. **(G)** *p < 0.05 vs. Ad-GFP group. ^#^*p* < 0.05 vs. Ad-GFP + DOX group. ^&^*p* < 0.05 vs. Ad-JMJD3 + DOX group. *n* = 3. DOX, doxorubicin; JMJD3, Jumonji domain-containing 3; SESN2, Sestrin2.

Sestrin2 was overexpressed by adenovirus or was knocked down by sgRNA in NRCMs. Consistent with our previous study (Wang et al., 2019), overexpression of SESN2 suppressed DOX-induced chromatin condensation and disorganized mitochondrial structure, and further decreased the protein level of cleaved caspase3 and BAX/Bcl2 ratio (Figures 7A–C). However, knockout SESN2 by targeting sgRNA obviously induced cardiomyocytes injury (Figures 7D–F). JMJD3 and SESN2 were co-expressed in NRCMs, to investigate the relationship between JMJD3 and SESN2 in DOX-induced chronic cardiotoxicity. Overexpression of SESN2 relieved JMJD3 overexpression or DOX-induced cardiomyocytes injury as indicated by decreased protein levels of cleaved caspse3 and BAX/Bcl-2 ratio, chromatic condensation and improved mitochondrial structure (Figures 7G–I). These data suggested that JMJD3 aggravated DOX-induced cardiotoxicity partly via inhibiting SESN2 transcription.

## Discussion

Doxorubicin is one of the most effectively anthracycline antitumor drugs in clinic (Carvalho et al., 2014), which is limited by its numerous side effects, especially cardiotoxicity (Olson and Mushlin, 1990). DOX-mediated cardiotoxicity is characterized by dilated cardiomyopathy, which affects the ventricles and atria, dilates heart muscle and chamber, and eventually lead to heart failure (Takemura and Fujiwara, 2007). The Dox-induced cardiotoxicity could be an arrhythmia, cardiomyopathy, left ventricular dysfunction, and congestive heart failure (Mitry and Edwards, 2016; Renu et al., 2018; D’Amario et al., 2020), which were partly consistent with clinical cardiomyopathy.

Jumonji domain-containing 3, a member of JMJD protein family, catalyzes the removal of methyl group from H3K27me3 and regulates gene transcription. JMJD3 participated a wide range of disease processes including immune diseases (Chen et al., 2018), cancer (Ramadoss et al., 2012), infectious diseases (Sun et al., 2019), aging related diseases (Salminen et al., 2014; Tamgue and Lei, 2017), and development diseases (Park et al., 2014). By regulating the expression of matrix metalloproteinase-3 (MMP-3) and MMP-9, JMJD3 was reported to mediate blood-spinal cord barrier disruption (Lee et al., 2016). JMJD3 mediated cell senescence via upregulating *p16INK4A* in kidney cancer (Shen et al., 2017). Our previous study revealed that JMJD3 played a role in pathological cardiac hypertrophy via regulating H3K27me3 in promoter region of β-MHC (Guo et al., 2018).

In this study, the mRNA of JMJD3 was increased in both human heart samples with dilated cardiomyopathy and DOX-induced chronic cardiomyopathy. RNAseq results revealed that the transcription of many other subtypes of JMJD protein family were also changed. However, the increase of JMJD3 was much higher than the others and our previous report also reported the important role of JMJD3 in cardiac hypertrophy (Guo et al., 2018). In this study, JMJD3 also aggravated DOX-induced chronic cardiomyopathy and cardiotoxicity, which revealed JMJD3 was a potential target in the therapeutic of cardiomyopathy and cardiac hypertrophy.

Sestrin2, a highly conserved stress-inducible metabolic protein, has been reported to repress oxidative or genotoxic stress (Budanov, 2011). SESN2 inhibits mTOR activity through direct association with AMPK or indirect transcriptional regulation (Pasha et al., 2017). We previously discovered that SESN2 protected against DOX-induced cardiotoxicity via rescuing mitophagy as well as improving mitochondrial function (Wang et al., 2019). In this study, the transcription of SESN2 was suppressed following chronic DOX stimulation at relative lower dose, which was inconsistent with previous results with acute DOX stimulation. Our data further validated the different pattern of SESN2 expression at different stages with DOX stimulation. In the acute stage, DOX might prefer to induce a quick degradation of SESN2 in proteasome pathway. However, DOX chronic stimulation at lower dose more likely depressed the transcription of SESN2.

It was largely unknown the potential mechanism of chronic DOX-stimulation induced depression on SESN2 transcription. Here, a key finding in our present study was that decreased expression of SESN2 was correlated with increased expression of JMJD3 during chronic DOX stimulation. Further result revealed that JMJD3 suppressed the transcription of SESN2 by decreasing H3K27me3 enrichment in promoter region of SESN2.

It is known that site-specific histone methylation is a crucial pathogenic mechanism in various diseases and conceivably represent therapeutic targets (Black et al., 2012; Jasiulionis, 2018). JMJD protein family catalyzes demethylation of methylated lysine and arginine residue of histone in mammalian cells (Krishnan and Trievel, 2016; Walport et al., 2016). JMJD3 specifically catalyzes the H3K27me3 to H3K27me2/1 and regulates gene expression (Salminen et al., 2014). JMJD3 has also been reported to demethylate non-histone protein retinoblastoma (RB) at the lysine 810 residue (K810) (Zhao et al., 2015). Interestingly, our study found that JMJD3 bind to the promoter region of SESN2 and decreased the enrichment of H3K27me3 in the promoter region which resulting in the transcriptional depression of SESN2.

Decreased H3K27me3 level at the promoter region always associated with transcriptional activation (Papait et al., 2020). However, changes of histone methylation not always strictly determine the transcriptional inhibition or activation of different target genes (Wang et al., 2016; Jasiulionis, 2018; Tabaei and Tabaee, 2019). One methyltransferase or demethylase may act as epigenetic silencer or activator at the same time (Wang et al., 2016). Here, we partly showed that histone demethylase JMJD3 might also be an epigenetic silencer during chronic DOX stimulation. However, JMJD3 directly or indirectly regulate the depression of SESN2 need further explanation.

## Conclusion

**FIGURE 8:**
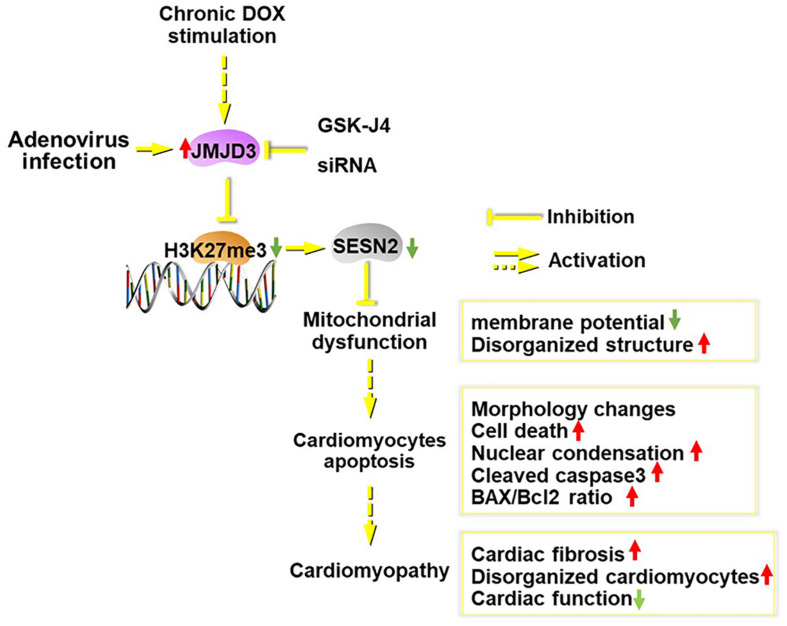
Scheme of this study. Chronic DOX stimulation or JMJD3 overexpression in NRCMs induced cardiomyocytes apoptosis, which was relieved by JMJD3 knockdown or GSK-J4 (enzyme activity inhibitor of JMJD3). The increased expression of JMJD3 was negatively correlated with the decreased expression of SEESN2 following DOX stimulation. Mechanically, JMJD3 catalyzed the demethylation of H3K27me3 in the promoter region of SESN2. Subsequently, the transcription of SESN2 was decreased and mitochondrial function was disrupted, which ultimately induced cardiomyocytes apoptosis and cardiomyopathy.

In summary (Figure 8), JMJD3 aggravated chronic DOX-stimulation induced cardiomyopathy via decreasing H3K27me3 enrichment in the promoter region of SESN2. Overexpression of SESN2 or suppression of JMJD3 effectively alleviated DOX-mediated cardiotoxicity. SESN2 mediated the effects of JMJD3 in cardiomyocytes and JMJD3 might act as an endogenous negative regulator for SESN2. In light of this, it might be a potential strategy to protect against cardiotoxicity via regulating JMJD3-SESN2 axis.

## Data Availability Statement

All datasets presented in this study are included in the article/Supplementary Material.

## Ethics Statement

The studies involving human participants were reviewed and approved by First Affiliated Hospital of Sun Yat-sen University. The patients/participants provided their written informed consent to participate in this study. The animal study was reviewed and approved by Experimental Animal Center of Sun Yat-sen University (Guangzhou, China).

## Author Contributions

PW and ZG performed the study, analyzed the data, and wrote and revised the manuscript. RL, QW, and SC contributed to the acquisition of data and data interpretation. ZL, JL, and ZZL contributed to manuscript preparation. ZW provided heart tissues from human with heart failure. JW, JL, and PL made the hypothesis and participated in the experimental design, and manuscript preparation. All authors approved the final version of the manuscript.

## Conflict of Interest

The authors declare that the research was conducted in the absence of any commercial or financial relationships that could be construed as a potential conflict of interest.

## Abbreviations

AMPK: AMP-activated protein kinase
BrdU: 5-bromodeoxyuridine
ChIP: chromatin immunoprecipitation
DMEM: Dulbecco’s modified Eagle’s medium
DCM: dilated cardiomyopathy
DOX: doxorubicin
HE: hematoxylin-eosin
i.p: intraperitoneally injection
IHC: immunohistochemistry
JMJD: Jumonji domain-containing
JMJD3: Jumonji domain-containing 3
K810: lysine 810 residue
KDM6b: lysine-specific demethylase 6b
LPS: lipopolysaccharide
MMP-3: matrix metalloproteinase-3
mTOR: mechanistic target of rapamycin
NS: normal saline
NRCMs: Primary-cultured of neonatal rat cardiomyocytes
PBS: phosphate buffered saline
PSR: Picro Sirius Red
RB: retinoblastoma
ROS: reactive oxygen species
SESNs: Serstrins
SESN2: Sestrin2
SD: Sprague-Dawley
TMRE: tetramethyl rhodamine methyl ester
β-MHC: β-myosin heavy chain.
