# Supplementary material for: Histone Demethylase JMJD3 Mediated Doxorubicin-Induced Cardiomyopathy by Suppressing SESN2 Expression
Source: Front Cell Dev Biol. 2020 Sep 29;8:548605. doi: 10.3389/fcell.2020.548605 (PMC7552667; doi:10.3389/fcell.2020.548605)
Supplement: Supplementary file 1 [file Data_Sheet_1.docx]

***Supplementary materials***

**1. Cell viability assay**

NRCMs were seeded onto 96-well plates and cell viability was assayed by using CCK8 assay kit (Ribio, Guangzhou, China). Following different treatment, 10 μL of CCK8 was added into the culture medium and 2 h later the absorbance was measured at 490 nm by using a microplate reader (Bio-Tek, Elx800, USA). The percent viability was defined as the relative absorbance of the treated cells versus the untreated control cells.

**Table S****1 SESN2 primer sequences for ChIP-PCR**

| **SESN2 promoter** | **Primer Sequences** |
| --- | --- |
| **Primer A** | Forward: 5'- GGCTACAGTTCTTGGACCCC-3' (-306 bp)  Reverse: 5'- TCCAGGAGAGGGGACAGATG-3' (-81 bp) |
| **Primer B** | Forward: 5'- CGGGAAAGAGCATCCAGTGT-3' (-850 bp)  Reverse: 5'- TCAAGTCCGTGACGCAATCA-3' (-672 bp) |
| **Primer C** | Forward: 5'- TACCATTGCTCAGGTGGCAG-3' (-1124 bp)  Reverse: 5'- CCAGCTCAGGGACACTTCAG-3' (-1022 bp) |
| **Primer D** | Forward: 5'- GACTGAGGCAAGACTGGGAC-3' (-1045 bp)  Reverse: 5'- GAGTCTGCCACCTGAGCAAT-3' (-934 bp) |

**2. Results**


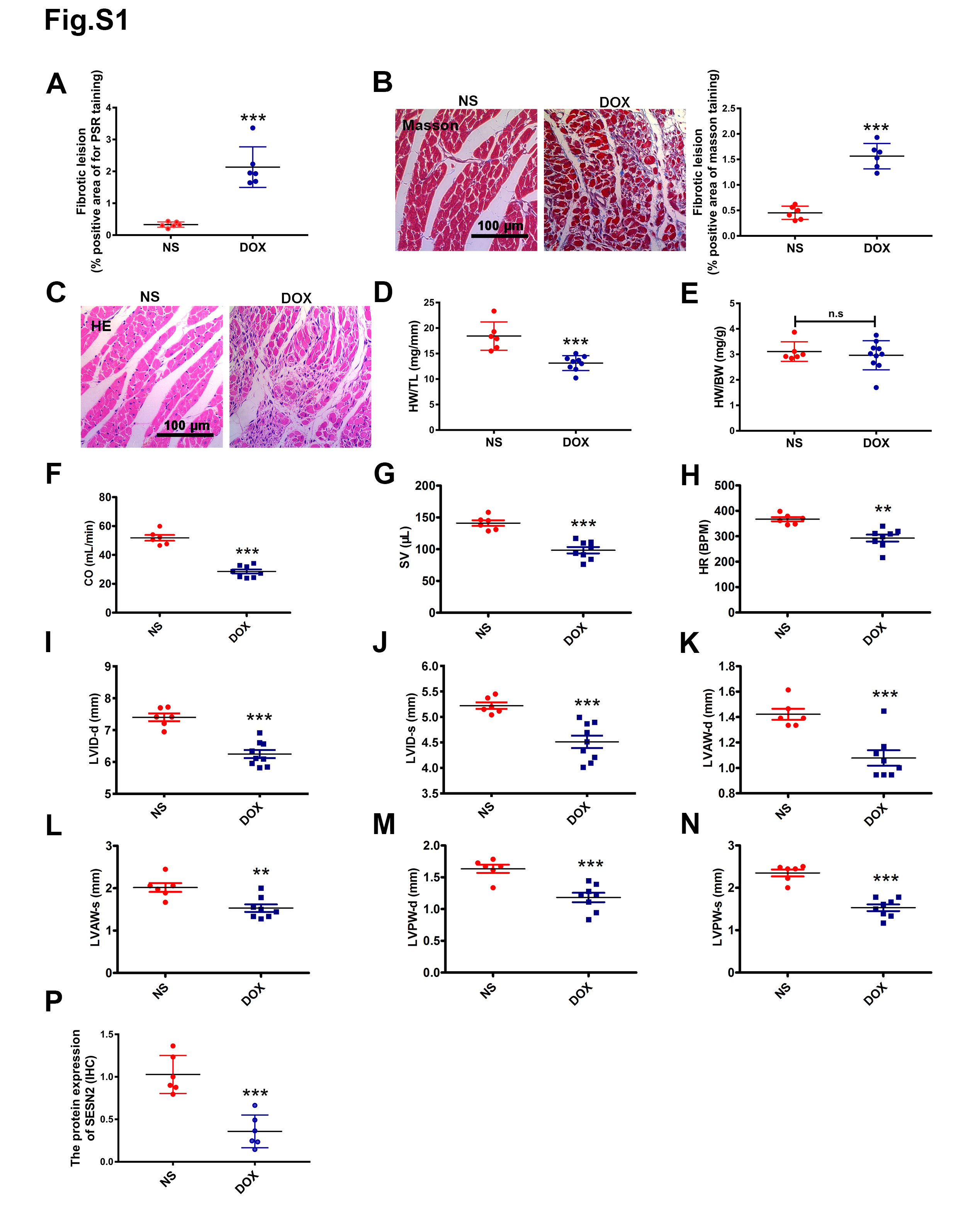


**Figure S1 DOX-induced rat cardiotoxicity model.** SD rats were treated with DOX 5 mg/kg/day or equal volume of normal saline (NS) at indicated day of 1, 5, 9 and 13, *i.p.*, *n* = 6 in NS group, *n* = 9 in DOX group. **(A)** Quantification of fibrosis of heart tissue from PSR staining by using Image J. **(B)** Fibrosis of heart tissue was indicated by masson staining and quantified by using Image J, scale bar: 100 μm. **(C)** Pathological changes of hearts tissues were detected by H&E staining, scale bar: 100 μm. **(D)** The ratios of HW/BW (mg/g) & HW/TL (mg/mm). **(E-N)** The CO (mL/min), SV (μL), HR (BPM), LVID-d (mm), LVAW-d (mm), LVAW-s (mm), LVID-s (mm), LVPW-d (mm), LVPW-s (mm) were measured. **(P)** The protein expression of SESN2 was quantified by using Image J. The data were presented as the mean ± SD. *^*^p <* 0.05, *^**^p <* 0.01, *^***^p <* 0.001 *vs.* NS group*.* Abbreviations: *DOX* doxorubicin, *SD* Sprague-Dawley, *NS* normal saline, *i.p* intraperitoneally injection, *HE* hematoxylin-eosin, *CO* Cardiac Output, *SV* Stroke Volume, *HR* Heart Rate, *LVAW-d* Left Ventricular end-diastolic Anterior Wall thickness, *LVAW-s* Left Ventricular end-systolic Anterior Wall thickness, *LVPW-d* Left Ventricular end-diastolic Posterior Wall thickness, *LVPW-s* Left Ventricular end-systolic Posterior Wall thickness, *LVID-d* Left Ventricular end-diastolic Internal Dimension, *LVID-s* Left Ventricular end-systolic Internal Dimension.


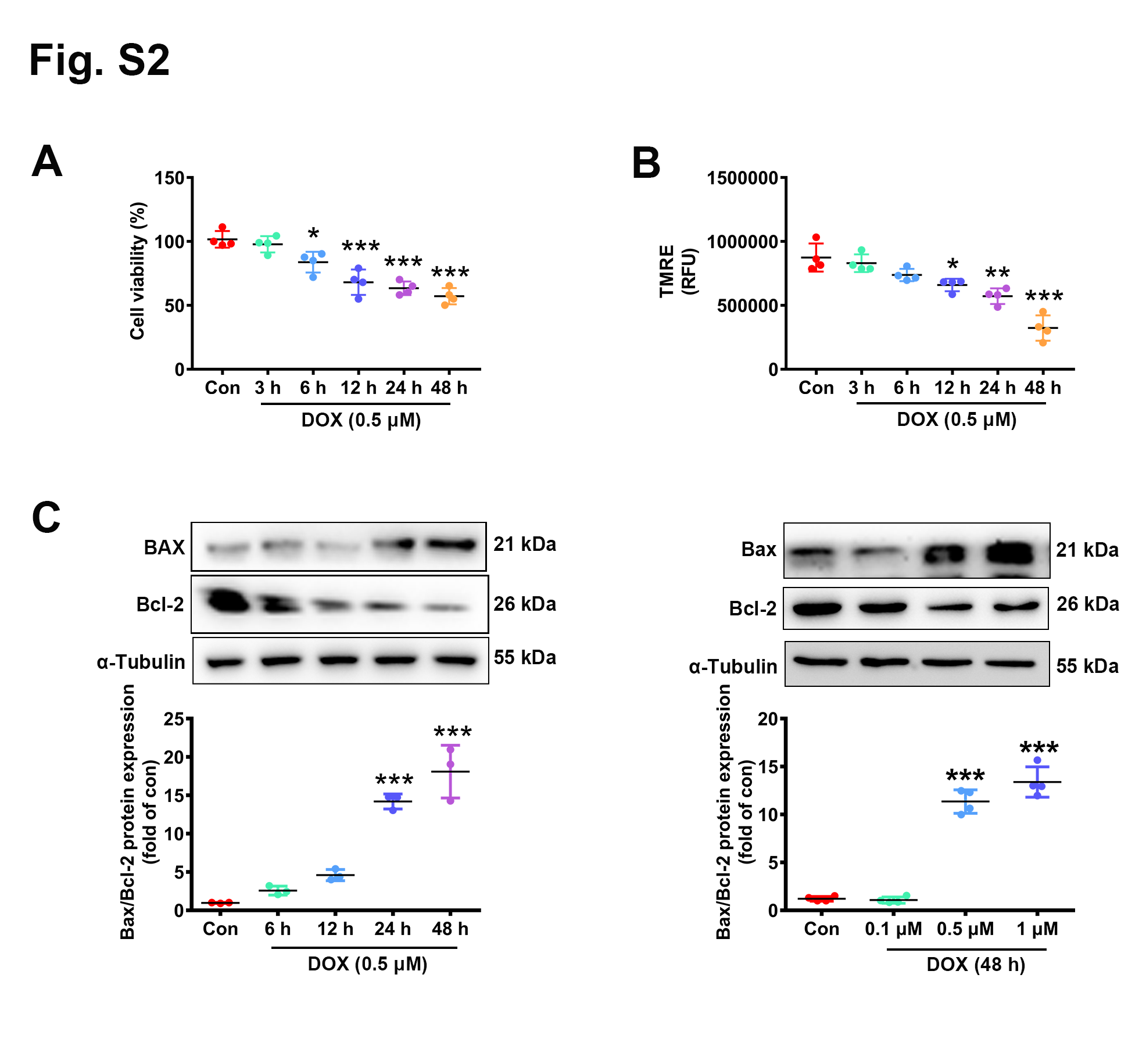


**Figure S2 DOX-induced cardiotoxicity model in NRCMs.** NRCMs were incubated by 0.5 μM DOX for indicated time points or with different concentrations of DOX for 48 h. **(A)** Cell viability changes was measured by using CCK8 assay. **(B)** Changes of mitochondrial membrane potential was quantified. **(C and D)** The ratios Bax/Bcl-2 were analyzed by Western blot. The data were presented as the mean ± SD. *^*^p* < 0.05*, ^**^p* < 0.01, *^***^p* < 0.001 *vs.* Con group*. n* = 3. Abbreviations: *DOX* doxorubicin, *NRCMs* Primary-cultured of neonatal rat cardiomyocytes.


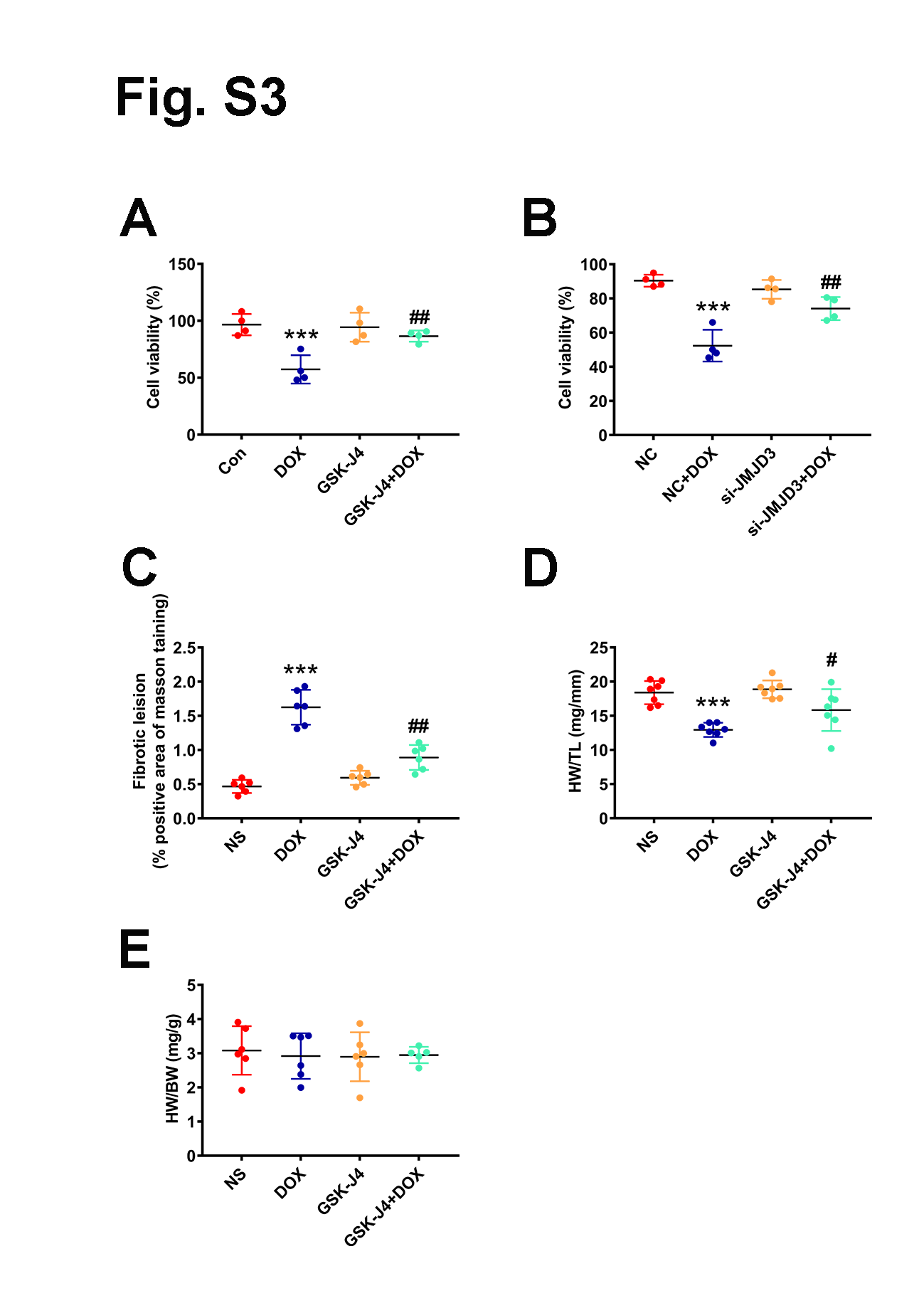


**Figure S3 Inhibition or knockout of JMJD3 relieved DOX-induced chronic cardiomyopathy.** GSK-J4 (5 μM) or siRNA targeting JMJD3 was co-treated with DOX (0.5 μM) were cotreated to NRCMs for 48 h. **(A and B)** Cell viability was assayed by CCK8. C57BL/6J mice were treated with GSK-J4 (10 mg/kg/day, *i.p*.) or equal volume of normal saline (NS) for 7 days. After that, the mice were injected with DOX at accumulative dose 28 mg/kg or equal volume of normal saline for three weeks. **(C)** Fibrosis of heart tissues was quantified by using ImageJ. (D and E) The heart weight to body weight ratio or heart weight to tibia length were analyzed. The data were presented as the mean ± SD.*^***^p <* 0.001 *vs.* control group; *^#^p* < 0.05*, ^##^p* < 0.01 *vs.* DOX group or NC + DOX group. *n* = 3. Abbreviations: *DOX* doxorubicin, *JMJD3* Jumonji domain-containing 3, *HW/BW* hearts weight/body weight, *HW/TL* heart weight/tibia length.


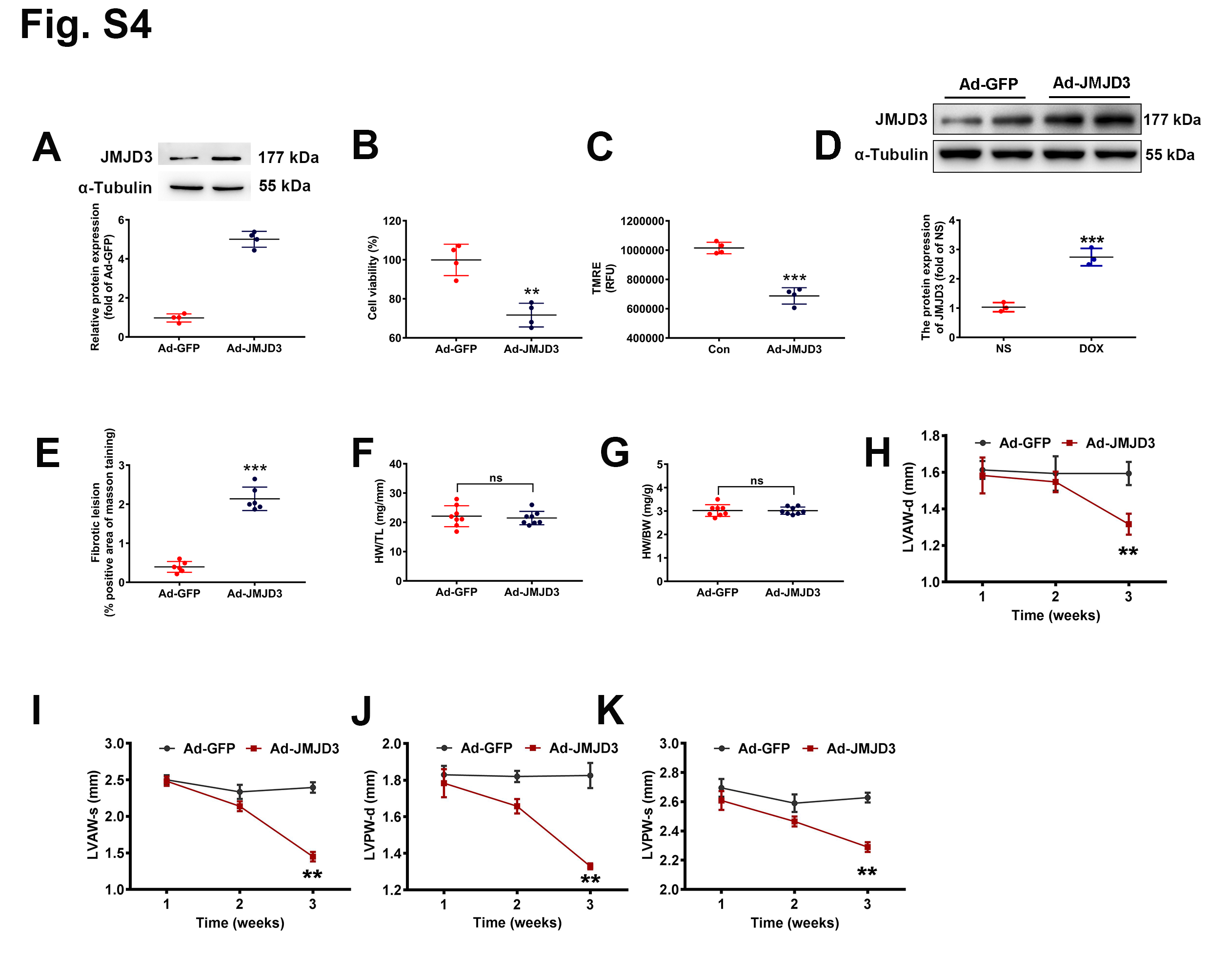


**Figure S4 JMJD3 overexpression aggravated DOX-induced cardiomyopathy *in vivo*.** Cardiomyocytes were infected by adenovirus to overexpress JMJD3. **(A)** The protein changes of JMJD3 was measured by western blot. **(B)** Cell viability was assayed by CCK8. **(C)** Mitochondrial membrane potential changes were quantified. The cardiac function was executed in 1, 2 and 3 weeks after the transfection of Ad-JMJD3 adenovirus or Ad-GFP. **(D)** The intracellular and extracellular protein level of JMJD3 were observed by Western blot. **(E)** The fibrosis of heart tissues was quantified. **(F and G)** HW/TL and HW/BW were measured. **(H-K)** The LVAW-d (mm), LVAW-s (mm), LVPW-d (mm), LVPW-s (mm) were measured. The data were presented as the mean ± SD. *^*^p <* 0.05, *^**^p <* 0.01, *^***^p <* 0.001 *vs.* control group*. n* = 3 (*in vitro*) or 8 (*in vivo*). Abbreviations: *DOX* doxorubicin, *JMJD3* Jumonji domain-containing 3, *LVAW-d* Left Ventricular end-diastolic Anterior Wall thickness, *LVAW-s* Left Ventricular end-systolic Anterior Wall thickness, *LVPW-d* Left Ventricular end-diastolic Posterior Wall thickness, *LVPW-s* Left Ventricular end-systolic Posterior Wall thickness, *HW/BW* hearts weight/body weight, *HW/TL* heart weight/tibia length.
